# Investigation of component alignment and patient factors for the risk of subsidence in cementless unicompartmental knee arthroplasty

**DOI:** 10.1007/s00402-025-06186-z

**Published:** 2026-02-02

**Authors:** Tomofumi Kinoshita, Kristian R. L. Mortensen, Christian Bredgaard Jensen, Kristine Ifigenia Bunyoz, Kirill Gromov, Anders Troelsen

**Affiliations:** 1 Department of Orthopaedic Surgery, Copenhagen University Hospital Hvidovre, Copenhagen, Denmark; 2 Department of Clinical Medicine, University of Copenhagen, Copenhagen, Denmark

**Keywords:** Unicompartmental knee arthroplasty, Mobile-bearing, Subsidence, Component position

## Abstract

**Introduction:**

This study identified the incidence and potential risk factors of tibial component subsidence in cementless unicompartmental knee arthroplasty (UKA) and evaluated its clinical impact.

**Methods:**

This retrospective cohort study analyzed 123 knees that underwent cementless Oxford mobile-bearing UKA. Anteroposterior and lateral radiographs were obtained preoperatively, immediately postoperatively, and at the first outpatient follow-up (3–6 months). Valgus subsidence was defined as a valgus change of > 2° in the tibial component angle between the immediate postoperative and first follow-up radiographs. The patients were categorized according to the presence or absence of subsidence. The alignment, component angles, and femoral component position relative to the tibial component in the coronal plane were compared between groups. Clinical outcomes were assessed using the Oxford Knee Score (OKS) at 3 months, 1 year, and 2 years.

**Results:**

Tibial component subsidence incidence was 4.8%. The immediate postoperative tibial component alignment was significantly less varus in the subsidence group than in the non-subsidence group (1.0 ± 1.9° vs. 3.3 ± 2.2°, *p* = 0.019). The femoral component was positioned significantly more laterally than the tibial component in the subsidence group (*p* = 0.009). The non-subsidence group was older and had a greater postoperative posterior tibial slope than the subsidence group; however, these differences were not statistically significant (*p* = 0.069 and 0.052, respectively). Clinical outcomes assessed by OKS were not significantly different between groups at any time point. No revision surgery owing to subsidence was required within 2 years.

**Conclusions:**

Subsidence occurred in 4.8% of cases and was associated with a relative lateral placement of the femoral component and less varus of tibial component alignment. A tibial cut with an appropriate varus angle may be protective. Given the small number of events, these findings should be interpreted as exploratory and inform future prospective studies.

**Supplementary Information:**

The online version contains supplementary material available at 10.1007/s00402-025-06186-z.

## Introduction

Over the past few decades, unicompartmental knee arthroplasty (UKA) clinical outcomes have become highly reliable, and the procedure is widely recognized as an effective treatment option for medial compartment osteoarthritis [1]. Initially, cemented UKA was predominantly performed; however, a high incidence of radiolucent lines (RLLs) was reported, raising concerns in clinical practice [2–5]. In recent years, cementless UKA has been increasingly adopted, which has a reduced RLL incidence compared with cemented fixation [4]. Additionally, comparative studies have reported that cementless UKA is associated with favorable clinical outcomes and lower revision rates [5].

Unlike cemented fixation, cementless UKA relies on direct bone–implant contact for initial stability, which may render it more sensitive to asymmetric load transfer. Studies using radiostereometric analysis (RSA) have demonstrated that early micromotion or subsidence of the tibial component can occur after cementless UKA [6]. The manifestation of microscopic subsidence on postoperative follow-up radiographs is a concern for surgeons [7]. Although the overall subsidence incidence is low, pain has been reported [7–9], making it a clinically important concern. Several investigators have attempted to clarify contributing factors, but the number of available reports remains limited [7–13], ; because of this, decisive risk factors for subsidence in cementless UKA have not yet been established. Previous studies have suggested the potential influence of patient-related factors such as age and bone quality, as well as intraoperative factors such as component alignment; however, these findings remain inconsistent and controversial. Although biomechanical hypotheses regarding femoral–tibial component positioning have been proposed [7], these relationships have not been systematically evaluated. This gap highlights the need for further radiographic investigation of femoral–tibial relative positioning.

The main objectives of the present retrospective study were to determine the incidence and potential risk factors of tibial component subsidence in cementless UKA, and to evaluate its impact on clinical outcomes. We hypothesized that biomechanical factors may influence the development of tibial component subsidence, and specifically that a relatively lateral positioning of the femoral component with respect to the tibial component in the coronal plane may increase the risk of valgus subsidence.

## Methods

### Patient selection

**Fig. 1:**
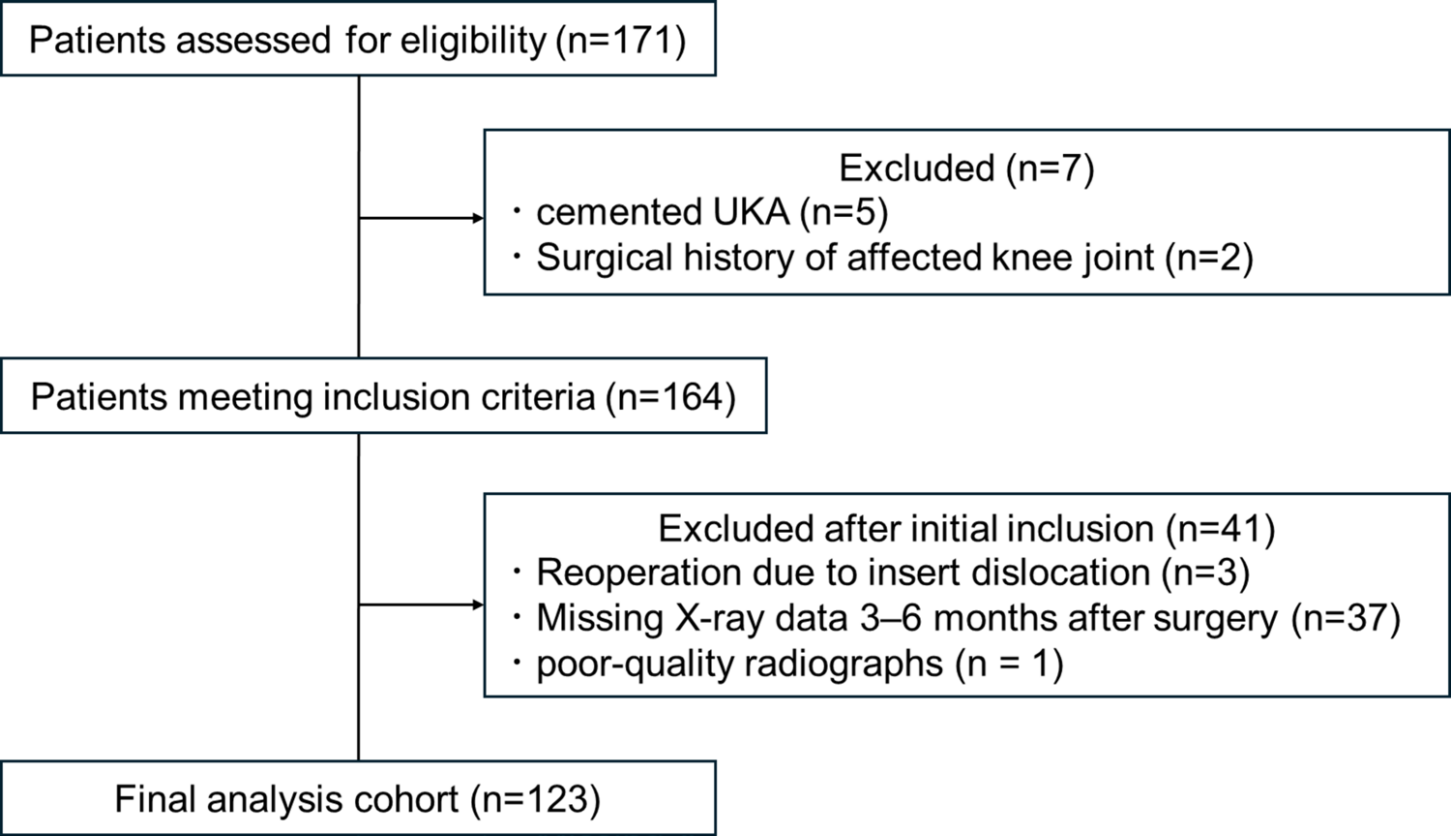
Flow diagram of patient inclusion and exclusion

This retrospective cohort study was conducted and reported in accordance with the STROBE (Strengthening the Reporting of Observational Studies in Epidemiology) guidelines. Patients with knee osteoarthritis who underwent primary cementless UKA between January 2018 and November 2019 in a single center and who provided informed consent (*n* = 171) were retrospectively identified and included. The exclusion criteria were history of ipsilateral knee surgery (*n* = 2), use of cemented UKA (*n* = 5), reoperation within 2 years of follow-up (insert dislocations, *n* = 3), absence of radiographic evaluation at our hospital within 6 months postoperatively (*n* = 37), and suboptimal radiographic imaging (*n* = 1). In total, 123 patients were included (Fig. 1). Of these, 55 patients underwent radiographic evaluation preoperatively as well as immediately, 3 months, and 1 year postoperatively; 68 had radiographic data available for all time points except for 1 year postoperatively.

Radiographic data were complete through 1 year postoperatively in 55 patients and through the 3–6-month follow-up in 68 patients; all patients had preoperative and immediate postoperative radiographs.

## Implant and surgery

All procedures were performed using the cementless Oxford Phase 3 mobile-bearing UKA (Zimmer Biomet, Oxford, UK) with microplasty instrumentation through a minimally invasive medial parapatellar approach without patellar eversion. The tibial cut was intended to be made perpendicular to the mechanical axis in the coronal plane and with a posterior slope of 7° in the sagittal plane. Femoral preparation was performed using an intramedullary guide, and the spherical femoral component was positioned as centrally as possible on the condyle. The tibial component was implanted to achieve optimal cortical coverage while avoiding anterior overhang and minimizing medial under- or overhang.

## Radiographic evaluation and outcome measures

**Fig. 2:**
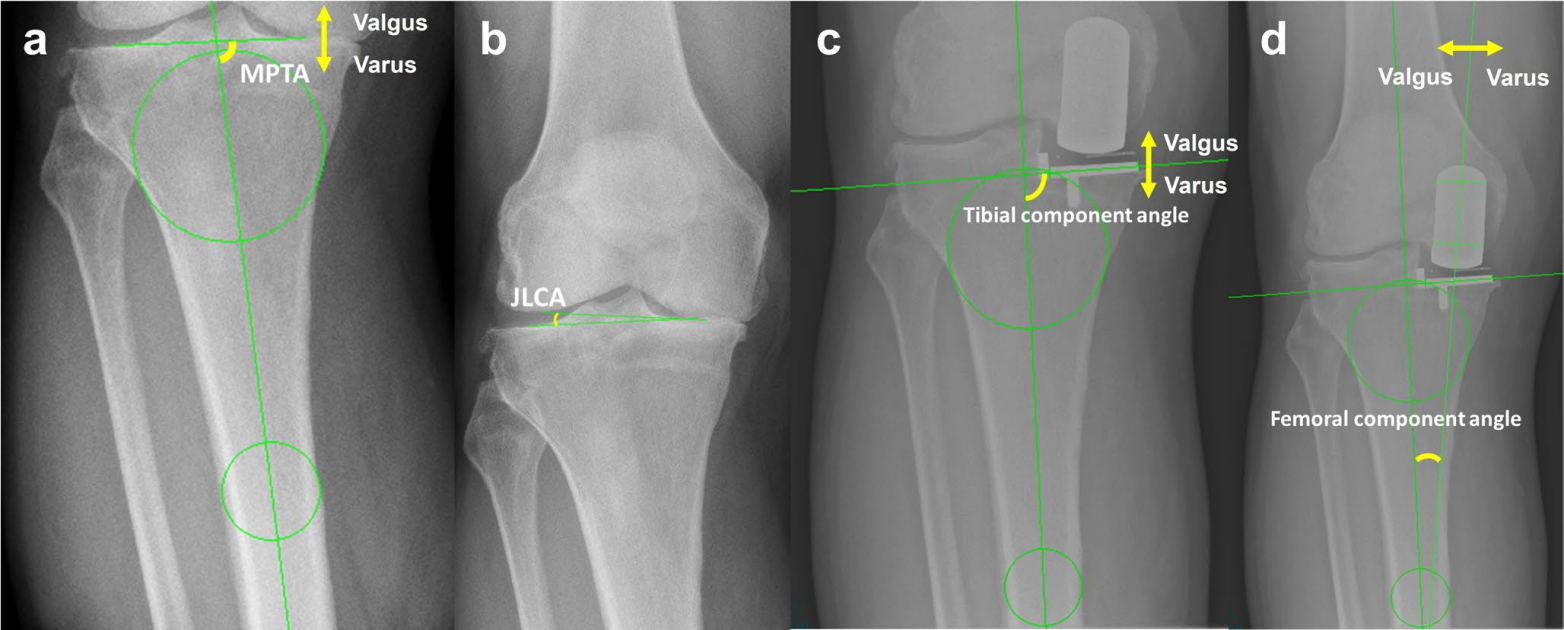
Radiographic measurement parameters. Representative radiographs showing the measurement methods. (a) Medial proximal tibial angle (MPTA): the angle between the tibial and tibial mechanical axes. (b) JLCA: joint line convergence angle. (c) Tibial component angle: angle between the tibial axis and the bisector line of the tibial component. (d) Femoral component angle: angle between the tibial axis and the bisector line of the femoral component. For consistency, valgus alignment on anteroposterior radiographs was defined as negative and varus alignment as positive

Short anteroposterior and lateral knee radiographs were obtained preoperatively, immediately postoperatively, at the first outpatient follow-up 3–6 months postoperatively, and at 1 year postoperatively. The femorotibial angle (FTA) and posterior tibial slope (PTS) were measured on short radiographs using a previously described method in which circles were drawn at the proximal and distal aspects of both the femur and tibia, and lines connecting their centers were defined as the respective mechanical axes [14]. The angle formed between these axes was defined as the FTA. Additionally, the medial proximal tibial angle (MPTA) and joint line convergence angle (JLCA) were assessed (Fig. 2). The FTA, femoral component alignment angle, tibial component alignment angle, and femoral component position relative to the tibial component in the coronal plane were measured on postoperative anteroposterior radiographs. The component alignment angles on the anteroposterior view were defined as the angle between the tibial axis (as defined above) and the bisector line of each component (Fig. 2). The PTS of the tibial component was measured laterally. For consistency, valgus alignment was defined as negative and varus alignment as positive on the anteroposterior radiographs, whereas the posterior slope was defined as positive and the anterior slope as negative on the lateral radiographs.

**Fig. 3:**
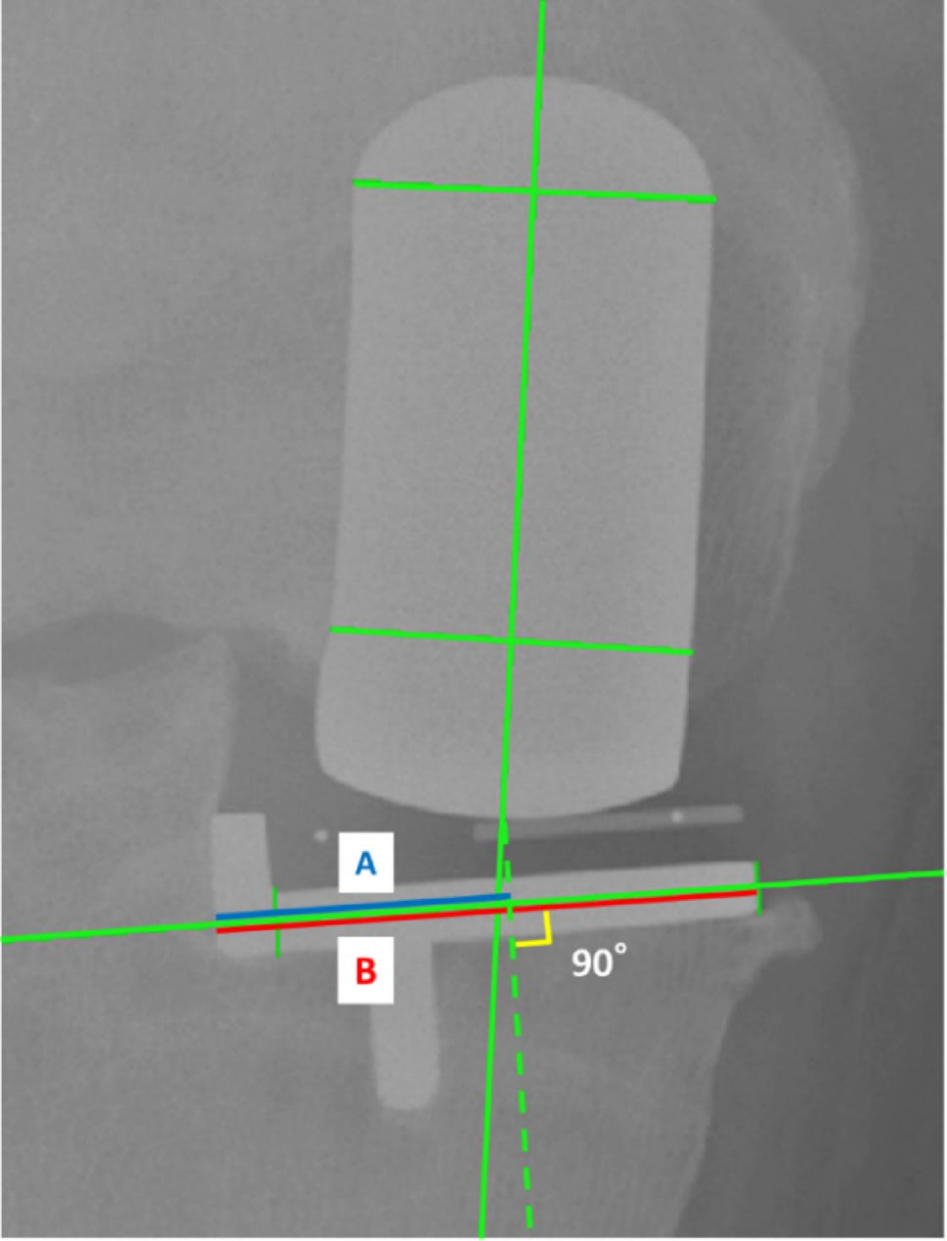
Measurement of the femoral component position relative to the tibial component in the coronal plane (contact point ratio)

The position of the femoral component relative to that of the tibial component in the coronal plane was evaluated using the contact point ratio (Fig. 3). A line was drawn connecting the midpoints of the proximal and distal aspects of the femoral component, and the most distal point of the femoral implant was identified along this line. From this point, a line perpendicular to the tibial component was drawn, and the intersection was defined as the contact point. The contact point ratio was calculated as the distance from the lateral edge of the tibial component to the contact point (A, Fig. 3) divided by the total width of the tibial component (B, Fig. 3), with 0 representing the most lateral edge and 1 representing the most medial edge. The contact point ratio was used to reflect the mediolateral relationship between the femoral and tibial components, which may influence mediolateral load transfer across the mobile bearing.

During the first outpatient follow-up, femoral and tibial component alignment angles were measured in the same manner. Valgus subsidence was defined as a change of > 2° toward valgus in the coronal tibial component angle between the immediate postoperative and first outpatient radiographs [8, 11]. All radiographic assessments were independently reviewed by two senior orthopedic surgeons, and complete agreement was reached. In this study, tibial component subsidence refers specifically to valgus subsidence in the coronal plane. In addition, postoperative clinical outcomes were evaluated using the Oxford Knee Score (OKS) at 3 months, 1 year, and 2 years after surgery. Radiographic measurements were performed without blinding to group allocation but were blinded to clinical outcomes.

A line was drawn connecting the midpoints of the proximal and distal aspects of the femoral component, and the most distal point of the femoral implant was identified along this line. From this point, a perpendicular line was drawn on the tibial component surface, and the intersection was defined as the contact point. The mediolateral location of the contact point is expressed as a ratio, with 0 indicating the most lateral edge and 1 indicating the most medial edge. The blue line (A) represents the distance from the lateral edge of the tibial component to the contact point and the red line (B) represents the total width of the tibial component. The A/B ratio is defined as the contact-point ratio.

### Statistical analysis

To evaluate intraobserver and interobserver reproducibility of all radiographic measurements, assessments were performed twice by one surgeon at intervals of more than 1 month and once by another examiner on ten knees randomly selected from the study group [8]. The intraclass correlation coefficients (ICCs; two-way mixed-effects model, consistency, single measures) between two repeated measurements by the same observer for FTA, MPTA, JLCA, the femoral component coronal alignment angle, tibial component coronal alignment angle, PTS, and the femoral component position relative to the tibial component were 0.953 (95% confidence interval [CI]: 0.934–0.967, *p* < 0.001), 0.831 (95% CI: 0.767–0.879, *p* < 0.001), 0.864 (95% CI: 0.811–0.903, *p* < 0.001), 0.944 (95% CI: 0.921–0.961, *p* < 0.001), 0.925 (95% CI: 0.895–0.947, *p* < 0.001), 0.885 (95% CI: 0.839–0.918, *p* < 0.001), and 0.972 (95% CI: 0.958–0.982, *p* < 0.001), respectively. The corresponding interobserver ICCs (two-way random-effects model with absolute agreement, single measures) between the two examiners for FTA, MPTA, JLCA, the femoral component coronal alignment angle, tibial component coronal alignment angle, PTS, and the femoral component position relative to the tibial component were 0.852 (95% CI: 0.530–0.960, *p* < 0.001), 0.817 (95% CI: 0.421–0.951, *p* = 0.001), 0.739 (95% CI: 0.285–0.927, *p* = 0.004), 0.967 (95% CI: 0.873–0.992, *p* < 0.001), 0.934 (95% CI: 0.752–0.983, *p* < 0.001), 0.790 (95% CI: 0.382–0.943, *p* = 0.001), and 0.913 (95% CI: 0.700–0.978, *p* < 0.001), respectively. This ICC analysis was performed on a randomly selected subset of knees and should be considered when interpreting the generalizability of the reproducibility estimates.

Non-parametric tests were applied for non-normally distributed data (as assessed by the Shapiro–Wilk test) or when the group sample size was small. Between-group comparisons of demographic variables, radiographic parameters, implant alignment, component size distributions, and clinical outcomes were performed using appropriate non-parametric or categorical tests (Mann–Whitney U test, Fisher’s exact test, or chi-squared test, as applicable). All statistical analyses were two-tailed, and a p-value < 0.05 was considered statistically significant. No a priori sample size calculations were performed because the incidence of the tibial component subsidence was low and remained uncertain. Because of the small number of subsidence events, multivariable regression analysis was not performed to avoid model overfitting and unstable estimates. No formal adjustment for multiple comparisons was applied because of the exploratory nature of the analysis and the limited number of events. Statistical analyses were performed using SPSS Statistics version 28.0 (IBM Corp., Armonk, NY, USA).

### Ethical considerations

This retrospective cohort study was conducted in accordance with the Declaration of Helsinki and Data gathering was approved by the Knowledge Centre on Data Protection Compliance, Capital Region of Denmark (P-2022-290) and patients provided informed consent for researchers to access their X-rays and electronic patient chart.

## Results

### Comparison of radiographic data and patient factors

**Fig. 4:**
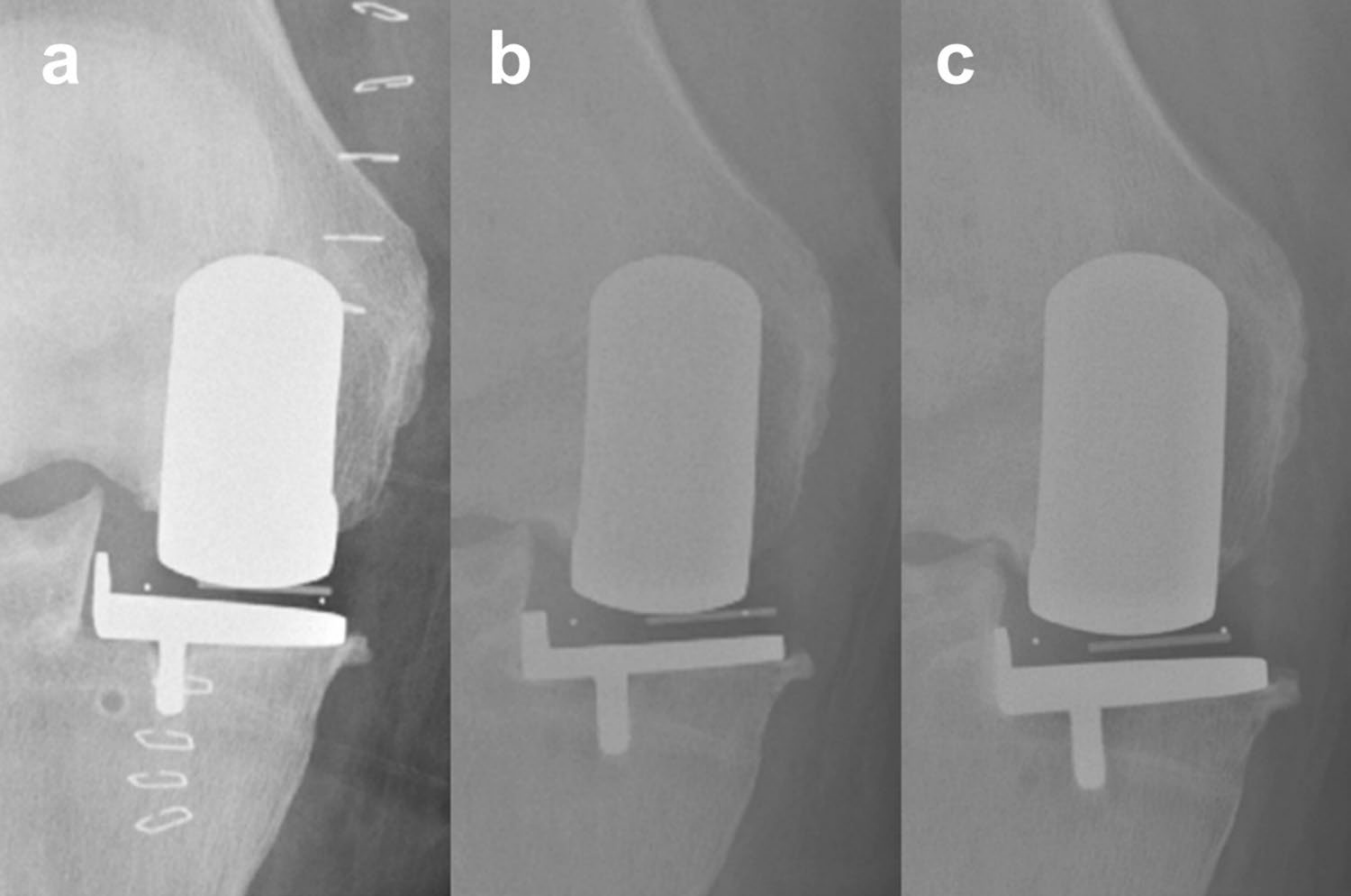
Representative radiographs of tibial component subsidence. (a) Immediate postoperative radiograph. (b) Radiograph at the first outpatient follow-up, showing valgus subsidence of the tibial component. (c) Radiograph at 1 year postoperatively, demonstrating no further progression of subsidence

The incidence of tibial component subsidence was 4.8% (6 of 123 cases; Fig. 4 and Supplementary Table 1). There were no statistically significant differences in preoperative radiographic parameters between the subsidence and non-subsidence groups (Table 1). Patients in the non-subsidence group were older; however, the difference was not statistically significant (*p* = 0.069). The postoperative tibial component alignment angle and femoral component position relative to the tibial component in the coronal plane differed significantly between groups (*p* = 0.019 and 0.009, respectively; Table 2). The postoperative PTS was greater in patients in the non-subsidence group, although the difference was not statistically significant (*p* = 0.052). Changes in FTA, MPTA, and PTS from pre-to postoperative did not differ significantly between groups (Table 2). There were no significant differences in the distribution of femoral or tibial component sizes between groups (*p* = 0.60 and 0.71, respectively).

**Table 1:** Preoperative patient characteristics

	Total Mean ± SD (range)	Subsidence group (range)	Non-subsidence group (range)	*p* value
Age (years)^‡^	66.8 ± 9.8 (39 to 93)	73.3 ± 9.1 (57 to 83)	66.5 ± 9.7 (39 to 93)	0.069
Sex (female/male)^†^	76/47	4/2	72/45	1.00
Height (cm)^‡^	171.1 ± 8.8 (155 to 197)	172.6 ± 7.3 (165 to 185)	171.1 ± 8.9 (155 to 197)	0.585
Weight (kg)^‡^	90.7 ± 20.5 (51 to 157)	80.1 ± 19.3 (62 to 112)	91.3 ± 20.5 (51 to 157)	0.157
Preoperative FTA (°)^‡^	1.6 ± 3.2 (-7 to 10)	2.5 ± 3.1 (0 to 7)	1.5 ± 3.1 (-7 to 10)	0.567
Preoperative MPTA (°)^‡^	85.3 ± 2.1 (80 to 90)	85.8 ± 2.8 (81 to 89)	85.3 ± 2.1 (80 to 90)	0.495
Preoperative JLCA (°)^‡^	5.0 ± 2.2 (0 to 12)	5.0 ± 1.6 (2 to 6)	5.0 ± 2.2 (0 to 12)	0.822
Preoperative posterior tibial slope (°)^‡^	8.5 ± 3.2 (1 to 14)	8.6 ± 5.5 (2 to 14)	8.5 ± 3.0 (1 to 14)	0.873

*SD* standard deviation, *FTA* femorotibial angle, *MPTA* medial proximal tibial angle, *JLCA* joint line convergence angle

†: Fisher’s exact test, ‡༚Mann–Whitney U test

**Table 2:** Postoperative patient characteristics and changes in radiographic characteristics from preoperative to immediate postoperative period after UKA between two groups

	Total Mean ± SD (range)	Subsidence group (range)	Non-subsidence group (range)	*p* value
Postoperative FTA (°)^‡^	-2.3 ± 2.5 (-8 to 5)	-2.8 ± 1.5 (-5 to -1)	-2.3 ± 2.6 (-8 to 5)	0.522
Postoperative femoral component angle (°)^‡^	6.3 ± 3.8 (-5 to 16)	6.6 ± 3.2 (3 to 11)	6.2 ± 3.8 (-5 to 16)	0.845
Postoperative tibial component angle (°)^‡^	3.1 ± 2.3 (-3 to 8)	1.0 ± 1.9 (-1 to 3)	3.3 ± 2.2 (-3 to 8)	0.019*
Postoperative posterior tibial slope (°)^‡^	7.8 ± 2.5 (1 to 14)	9.5 ± 1.5 (8 to 12)	7.7 ± 2.5 (1 to 14)	0.052
Femoral component position relative to the tibial component in coronal plane^‡^	0.54 ± 0.10 (0.33 to 0.78)	0.43 ± 0.09 (0.34 to 0.57)	0.55 ± 0.10 (0.33 to 0.78)	0.009*
Changes in FTA (pre-op to immediate post-op) (°)^‡^	-4.0 ± 2.9 (-11 to 6)	-5.3 ± 2.1 (-8 to -3)	-3.9 ± 3.0 (-11 to 6)	0.242
Change in MPTA (pre-op to immediate post-op) (°)^‡^	-1.4 ± 3.2 (-7 to 9)	-3.1 ± 2.1 (0 to 6)	-1.3 ± 3.2 (-7 to 9)	0.119
Changes in posterior tibial slope (pre-op to immediate post-op) (°)^‡^	-0.7 ± 3.9 (-10 to 10)	1.2 ± 5.5 (-4 to 8)	-0.8 ± 3.8 (-10 to 10)	0.465

The femoral component position relative to the tibial component in coronal plane is presented to two decimal places because it represents a normalized continuous variable ranging from 0 to 1

*UKA* unicompartmental knee arthroplasty, *SD* standard deviation, *FTA* femorotibial angle, *MPTA* medial proximal tibial angle

‡Mann–Whitney U test

*Significantly different between groups (*p* < 0.05)

### Clinical comparison

**Fig. 5:**
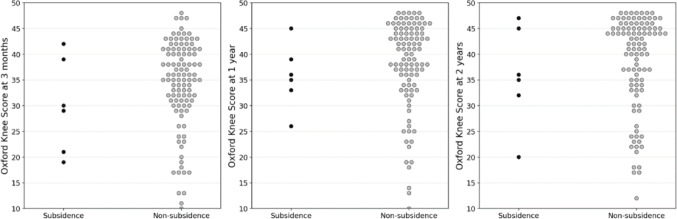
Oxford Knee Score (OKS) at 3 months, 1 year, and 2 years postoperatively in the subsidence and non-subsidence groups. Dot plots of the Oxford Knee Score (OKS) at 3 months, 1 year, and 2 years postoperatively in the subsidence and non-subsidence groups. Each dot represents a single patient

Clinical outcomes, including OKS at 3 months, 1 year, and 2 years postoperatively, showed no significant differences between the subsidence and non-Subsidence groups (30.0 vs. 34.3 at 3 months, *p* = 0.216; 35.6 vs. 38.0 at 1 year, *p* = 0.232; and 35.8 vs. 38.5 at 2 years, *p* = 0.431; Fig. 5). Three cases of dislocation were observed during the 2-year follow-up period (Fig. 1). None of these cases showed subsidence, and all were successfully revised with isolated bearing exchange. The temporal relationship between radiographic tibial component subsidence and clinical symptoms was not systematically assessed in this study.

### Longitudinal radiographic changes

**Fig. 6:**
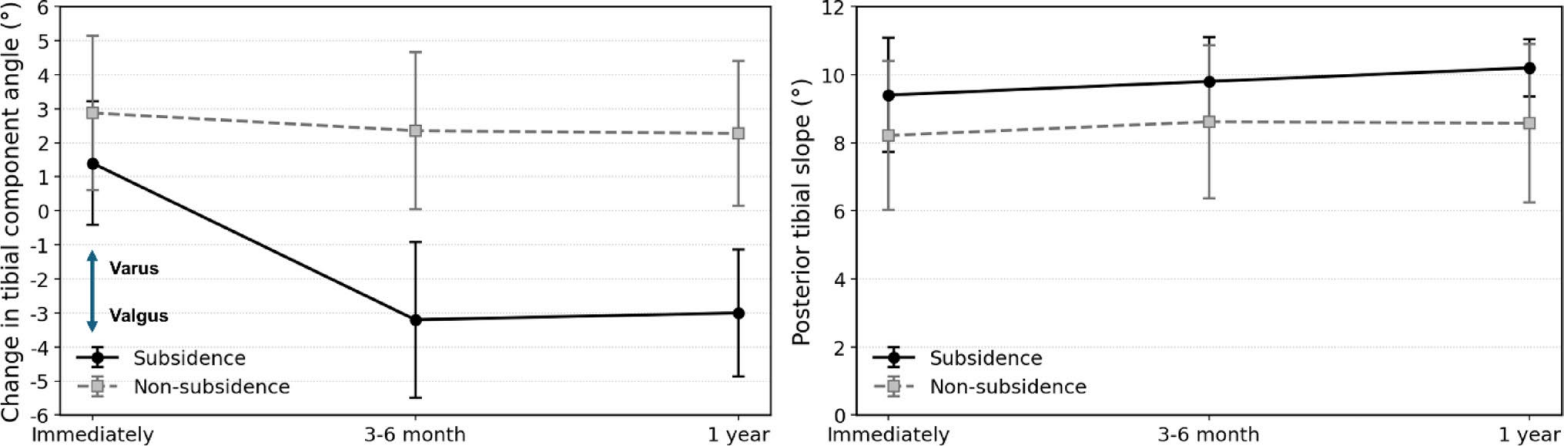
Difference of longitudinal changes in tibial component angle and posterior tibial slope between the subsidence and non-subsidence groups. The mean change in the tibial component angle (°) with standard error bars is shown for the subsidence (solid line) and non-subsidence (dashed line) groups immediately postoperatively, at the first outpatient follow-up (3–6 months), and at 1 year. In the subsidence group, valgus changes in the tibial component angle were mainly observed at 3–6 months with no further progression at 1 year. Error bars indicate standard deviation (SD)

The mean change in tibial component angle at the first outpatient follow-up in the subsidence group (*n* = 6) was − 4.6 ± 1.0°, with an absolute valgus change ranging from − 3° to − 6°. In the subsidence group, radiographic analysis of the 55 patients with complete longitudinal follow-up demonstrated that tibial component subsidence occurred at the first outpatient follow-up (3–6 months), with no further progression observed at 1 year (Fig. 6). No cases showed a marked change in the PTS, suggesting subsidence in the sagittal plane.

## Discussion

The incidence of tibial component subsidence after cementless Oxford mobile-bearing UKA was 4.8%, consistent with rates reported in the literature. Postoperative tibial component alignment in the coronal plane differed significantly between groups. The non-subsidence group showed slight varus alignment, whereas the subsidence group was closer to neutral. The femoral component was also located more laterally relative to the tibial component in the subsidence group. Although causality cannot be established, this pattern is consistent with a biomechanical hypothesis of asymmetric load transfer across the mobile bearing, which may increase stress on regions with lower bone resistance and predispose to valgus subsidence. This proposed mechanism is in line with previously reported hypotheses and clinical observations in cementless UKA [7].

Only a few comparative studies have examined factors associated with the occurrence of tibial component subsidence, making this an area with limited evidence. Kamenaga et al. reported subsidence in 6 of 120 cases (5%), although no differences in mean age, T-score, or bone mineral density were observed between groups [8]. In contrast, Gallant et al. reported subsidence in 8 of 97 cases (8.2%); there were lower T-scores and a higher proportion of female patients in the subsidence group, suggesting that bone quality and sex may contribute to this risk [11]. Hefny et al. reported subsidence in 4 of 158 cases (2.5%), all in patients aged ≥ 70 years, directly implicating advanced age as a contributing factor [10]. The age difference between groups was not statistically significant. This numerical difference should be interpreted cautiously given the small event number. Advanced age may still be clinically relevant and warrants evaluation in future studies, particularly in relation to bone quality.

In addition to patient-related factors such as age and bone quality, intraoperative tibial component alignment has also been implicated to have an impact on the risk of subsidence. Gallant et al. emphasized that larger corrections of tibial alignment from severe preoperative varus to postoperative neutral or valgus may increase the lateral load and contribute to subsidence [11]. Liddle et al. reported that valgus-directed loading of the tibial component can lead to subsidence in the coronal plane [7]. In our study, there were significant group differences in the postoperative tibial component alignment angle (*p* = 0.019), with the subsidence group being closer to neutral and the non-subsidence group tending to remain slightly varus. These findings suggest that valgus-oriented tibial implantation increases the lateral load on the tibial component, promoting subsidence. This may be related to the fact that a valgus-oriented tibial cut results in deeper central resection, where the bone becomes weaker. Moreover, in knees with medial OA, valgus tibial component placement has been reported to increase the risk of tibial fracture, and particular caution is warranted [15].

In addition to coronal alignment, sagittal alignment is considered an important factor. Several authors have suggested that excessive reduction and malalignment of the PTS may compromise tibial component fixation and increase revision risk [16–18]. Kurihara et al. reported anterior collapse of the tibial component in 5 of 182 cases, with 20% requiring revision to total knee arthroplasty [17]. In an RSA study, Horsager et al. reported a correlation between the PTS and component micromotion after UKA [6]. In our cohort, postoperative PTS was numerically greater in the subsidence group (9.5° ± 1.5°) than in the non-subsidence group (7.7° ± 2.5°). However, this difference was not statistically significant (*p* = 0.052). Given the limited statistical power, this numerical difference should be interpreted with caution. Although no definitive association can be confirmed, a potential influence of PTS on subsidence risk cannot be excluded and warrants further investigation in adequately powered studies.

Several studies have highlighted the relative positioning of the femoral component with respect to the tibial component. Kamenaga et al. reported that subsidence was associated with intraoperative factors, including the mediolateral position of the tibial and femoral components [8]. Gallant et al. similarly found that subsidence occurs when the tibial component is positioned more medially, and the femoral component is positioned more laterally relative to the tibia [11]. Liddle et al. explained the mechanism by which the lateral placement of the femoral component relative to the tibia could lead to bearing impingement, tilting, and valgus subsidence [7]. In our study, there were significant group differences in the position of the femoral component relative to the tibial component in the coronal plane (*p* = 0.009), supporting the notion that positioning of the femoral component may be a critical contributor to subsidence. Although the contact point ratio was measured postoperatively, it reflects the mediolateral femoral–tibial relationship established intraoperatively and may be useful for avoiding excessive lateralization of femoral component.

The effect of subsidence on pain and function remains unclear. Kamenaga et al. reported poorer outcomes at 3 months in the subsidence group [8], and Liddle et al. noted pain and stiffness in all subsidence cases [7]. Saleh et al. reported that most subsidence cases with all-polyethylene tibial components were painful, with some requiring revision [9]. Conversely, Hefny et al. found that three of four subsidence cases showed no progression with relatively favorable functional outcomes [10]. In our study, no revisions were required because of subsidence, and the overall functional outcomes did not differ significantly between the subsidence and non-subsidence groups at 3 months, 1 year, or 2 years postoperatively. These findings support conservative treatment with off-loading. In the absence of progressive radiographic changes or worsening clinical symptoms, an initial strategy of observation with activity modification appears reasonable. Close monitoring is essential, particularly in patients with persistent pain. Although no difference in OKS was observed, patient-reported outcome measures such as the OKS may not fully capture subtle early mechanical symptoms.

This study had several limitations. First, the number of cases with tibial component subsidence was very small, reflecting its low incidence; however, this severely limited the statistical power and the precision of effect estimates. Accordingly, the observed associations should be interpreted with caution and considered hypothesis-generating rather than confirmatory. Therefore, non-significant differences in OKS may reflect limited statistical power rather than true equivalence between groups. Second, several important variables that may confound the risk of tibial component subsidence were not assessed in this study, including bone mineral density and bone quality, preoperative osteoarthritis severity, body mass index, and postoperative activity level. Each of these factors has been suggested to influence implant fixation and load tolerance, and their absence may have affected the observed associations. Third, the retrospective cohort design introduced the possibility of selection and information biases. Radiographic assessment relied on plain radiographs rather than full-length standing or computed tomography (CT) imaging, limiting evaluation of rotational component positioning, cortical coverage, and the implant–bone interface. Moreover, patients without adequate radiographic follow-up at 3–6 months (*n* = 37) were excluded, which may have introduced selection bias. However, comparison of clinical outcomes showed no significant differences in OKS at 3 months or 1 year between patients with and without 3–6-month radiographic follow-up, suggesting that systematic exclusion of patients with poorer clinical outcomes is unlikely. Finally, this study evaluated only a single cementless UKA implant design. Therefore, the present findings may be implant-specific. Multiple statistical comparisons were also performed in an exploratory context without formal adjustment, increasing the risk of type I error. Overall, these limitations underscore that the present findings should be interpreted as exploratory and hypothesis-generating.

Despite these limitations, this study provides valuable insight into a rarely reported complication following cementless UKA. To date, only a few publications have compared tibial component subsidence using a defined comparator group, and all have been limited by small sample sizes. The present study adds to this scarce body of evidence by providing preliminary data to guide future hypothesis-driven research. Collectively, the limited number and size of existing studies highlight the need for prospective multicenter studies with larger cohorts, computed tomography based assessment, and longer-term follow-up to confirm risk factors and clarify the clinical significance of tibial component subsidence after cementless UKA.

## Conclusion

Subsidence occurred in 4.8% of cases and was associated with a relative lateral placement of the femoral component and less varus of tibial component alignment. A tibial cut with an appropriate varus angle may be protective. Given the small number of events, these findings should be interpreted as exploratory and inform future prospective studies.

## Supplementary Information

Below is the link to the electronic supplementary material.

Supplementary Material 1

## Data Availability

The datasets generated and analyzed in this retrospective study (radiographic measurements and PROM data) are not publicly available due to patient privacy and institutional regulations. Data are available from the corresponding author upon reasonable request and with permission from the institution.
