# Supplementary material for: Investigation of component alignment and patient factors for the risk of subsidence in cementless unicompartmental knee arthroplasty
Source: Arch Orthop Trauma Surg. 2026 Feb 2;146(1):38. doi: 10.1007/s00402-025-06186-z (PMC12864263; doi:10.1007/s00402-025-06186-z)
Supplement: Supplementary file 1 — Supplementary Material 1 [file 402_2025_6186_MOESM1_ESM.docx]

Supplementary Table 1. Clinical and radiographic characteristics of the cases with tibial component subsidence

|  | **Case 1** | **Case 2** | **Case 3** | **Case 4** | **Case 5** | **Case 6** |
| --- | --- | --- | --- | --- | --- | --- |
| Age | 57 | 80 | 83 | 75 | 74 | 71 |
| Preoperative FTA (°) | 1 | 1 | 7 | 0 | 6 | 0 |
| Postoperative FTA (°) | -3 | -2 | -1 | -4 | -2 | -5 |
| Postoperative tibial component angle (°) | 2 | 0 | -1 | 3 | 3 | -1 |
| Postoperative posterior tibial slope (°) | 9 | 10 | 12 | 8 | 8 | 10 |
| Femoral component position relative to the tibial component in coronal plane | 0.35 | 0.46 | 0.39 | 0.57 | 0.34 | 0.45 |

FTA, femorotibial angle.

The femoral component position relative to the tibial component in coronal plane is presented to two decimal places because it represents a normalized continuous variable ranging from 0 to 1.
